# Interaction of Herbal Compounds with Biological Targets: A Case Study with Berberine

**DOI:** 10.1100/2012/708292

**Published:** 2012-11-13

**Authors:** Xiao-Wu Chen, Yuan Ming Di, Jian Zhang, Zhi-Wei Zhou, Chun Guang Li, Shu-Feng Zhou

**Affiliations:** ^1^Department of General Surgery, The First People's Hospital of Shunde, Southern Medical University, Shunde, Guangdong 528300, China; ^2^School of Health Sciences and Health Innovations Research Institute, RMIT University, Bundoora, VIC 3083, Australia; ^3^Department of Surgery, The Third Hospital of Nanchang, Jiangxi, Nanchang 330009, China; ^4^Department of Pharmaceutical Science, College of Pharmacy, University of South Florida, 12901 Bruce B. Downs Boulevard, MDC 30, Tampa, FL 33612, USA

## Abstract

Berberine is one of the main alkaloids found in the Chinese herb Huang lian (*Rhizoma Coptidis*), which has been reported to have multiple pharmacological activities. This study aimed to analyze the molecular targets of berberine based on literature data followed by a pathway analysis using the PANTHER program. PANTHER analysis of berberine targets showed that the most classes of molecular functions include receptor binding, kinase activity, protein binding, transcription activity, DNA binding, and kinase regulator activity. Based on the biological process classification of *in vitro* berberine targets, those targets related to signal transduction, intracellular signalling cascade, cell surface receptor-linked signal transduction, cell motion, cell cycle control, immunity system process, and protein metabolic process are most frequently involved. In addition, berberine was found to interact with a mixture of biological pathways, such as Alzheimer's disease-presenilin and -secretase pathways, angiogenesis, apoptosis signalling pathway, FAS signalling pathway, Hungtington disease, inflammation mediated by chemokine and cytokine signalling pathways, interleukin signalling pathway, and p53 pathways. We also explored the possible mechanism of action for the anti-diabetic effect of berberine. Further studies are warranted to elucidate the mechanisms of action of berberine using systems biology approach.

## 1. Introduction

The majority of clinical drugs achieve their effect by binding to a cavity and regulating the cavity, of its protein targets [1]. In general, drugs act on four main types of regulatory proteins that mediate the actions of hormones, neurotransmitters, and autacoids. These four types of regulatory proteins are carriers, proteins, ion channels, and receptors [2]. Certain characteristics are expected for therapeutic targets [3]. A potential target needs only not to be druggable but also linked to disease, most preferably playing critical and inimitable roles in disease state. Binding sites are to have certain structural and physiochemical properties to accommodate high-affinity site-specific binding and subsequent regulation of protein activity by drugs. They are not significantly involved in other important biological processes to avoid potential side effects. Useful information about these targets may be investigated by analysing their sequence properties, protein families, structural folds, biochemical classes, similarity proteins, gene location in the human genome, and associated pathways [4]. This information can be potentially useful in derivation of rule and developing predictive tools in the search for druggable and potential targets [4].

The number of molecular targets acted on by current drug therapy is still in dispute. In 1996, Drews and Ryser identified a total of 483 drug targets addressed by drug therapy [5, 6]. Approximately 45% are cell membrane receptors, 28% are enzymes, and the remaining classes comprise hormones (11%), ion channels (5%), nuclear receptors (2%), and DNA (2%). About 7% of the targets are not known biochemically. Later, Hopkins and Groom challenged this figure and suggested that “rule-of-five” compliant drugs acted primarily through only 120 underlying molecular targets [3, 7]. However, the statistical analysis of disease genes and related proteins suggested that the total number of the estimated potential targets in the human genome ranges from 600 to 1,500 [3]. In the meantime, another report showed the estimated total number of distinct targets is in the range of 1,700–3,000 [8]. Chen et al. reported targets collected in the Therapeutic Target Database [9] is 997 distinct proteins, 1,494 distinct protein subtypes, and 41 nucleic acids, which are only targeted by at least one marketed drug and 1,267 research targets, which are only targeted by investigational agents that are not approved for clinical use at present [4]. Targets for neoplasm diseases, circulatory system diseases, infectious diseases, and nervous system and sense organs disorders constitute the largest number of targets [1]. An increase in target numbers is made possible by advances in genomics, proteomics, better molecular understanding of diseases, and increased effort in the exploration of new therapeutic targets as well as increased knowledge of unknown or unreported targets of previous existing drugs. An improvement in technology for target identification and validation also contributes greatly.

Chinese herbal medicine (CHM) has always been an integral part of traditional Chinese medicine (TCM), which has been practiced in the east for thousands of years. Chinese herbs are usually in the forms of dried whole plants or parts of the plants (roots, leaves, body, etc.); sometimes shells and even minerals are used. Chinese herbs are often used in a compound formula, consisting of several different herbs hosting different roles according to the principle of Jun-Chen-Zuo-Shi described by the ancient Chinese. Each of Jun, Chen, Zuo, and Shi function together to harmonise the body, with Shi (courier) herbs are included in many formulae to ensure that all components in the prescription are well absorbed and to help to deliver or guide them to the target organs [10]. On some level, the guiding function of Shi herbs relates to modern drug delivery techniques, guiding the drug compound to target tissues. In the modern world, complementary medicine has gained vast popularity in the West. There has been increased use of herbal medicine to manage chronic diseases and promote wellbeing, in countries such as Australia, New Zealand, USA, and Europe [11]. Reports show that 18.9% of the American population used natural products in the precedent year [12]. This increase in popularity is closely related to its proven effectiveness in clinical practice over the past centuries. To date, more than 11,000 species of plants are used medicinally and about 300 are commonly used [13].

Despite its widespread use, CHM is associated with high levels of uncertainty. This is mainly due to lack of evidence, base of efficacy, targets, and safety data. During the process of therapeutic drug development, owing to the preselection of targets, researchers have a basic if not full understanding of which molecular structures the drug will react with or which biological pathway in the body it might alter. Knowledge on molecular interactions and modulations of the drug is anticipated and researched on. However, this is not the case for CHM. There is no preselection of molecular targets in the body but CHM has been used for thousands of years and is proven to be effective. The exact mechanism of the herbs actions is yet to be elucidated.

The proven clinical efficacy of some herbal medicines is considered to be due to the interaction of pharmacologically active components from the herbs with molecular targets in the body. Similar to synthetic drugs, active compounds of herbal medicine may bind to and undergo interactions with molecular structures or herbal targets to produce therapeutic or adverse effects. However, there is a lack of understanding of how CHMs exert their biological and clinical effects at a molecular level, which impedes development of CHMs and the incorporation of CHMs into mainstream medicine in the West.

**Figure 1:**
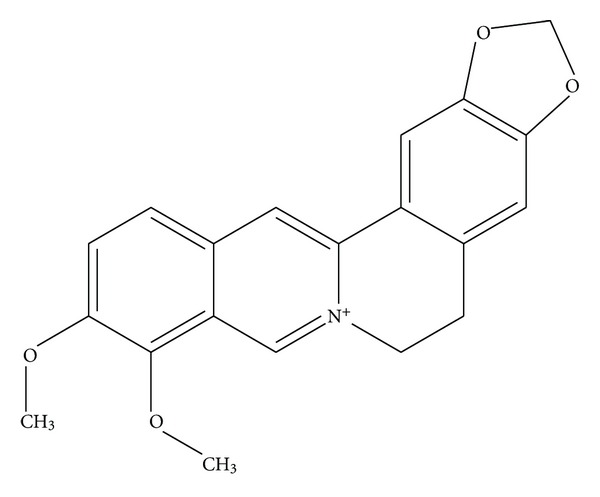
Chemical structure of berberine.

Berberine (Figure 1, molecular formula C_20_H_19_NO_5_ and a molecular weight of 353.36) is an isoquinoline alkaloid found in many medicinal plants [14]. It is a major constituent of many medicinal plants of families Papaveraceae, Berberidaceae, Fumariaceae, Menispermaceae, Ranunculaceae, Rutaceae, and Annonaceae [15]. It is present in *Hydrastis canadensis* (goldenseal), *Coptis chinensis* (Coptis or goldenthread), *Berberis aquifolium* (Oregon grape), *Berberis vulgaris* (barberry), and *Berberis aristata* (tree turmeric). The berberine alkaloid can be found in the roots, rhizomes, and stem bark of the plants. Berberine is one of the main alkaloids found in the Chinese herb Huang Lian (*Rhizoma coptidis*) [16]. Huang Lian has traditionally been used to treat diarrhoea and diabetes. In China, berberine has been manufactured into the over-the-counter drug Huang Lian Su Pian, also known as Coptis Extract Tablets for the treatment of traveler's diarrhoea [14, 17]. In recent years, there has been a growing interest in the pharmacological activities of berberine and many studies have been carried out to elucidate the mechanisms of action of berberine. This study aims to review molecular targets of berberine based on *in vitro* studies. Berberine has shown to have good hypoglycaemic effects, so we also reviewed the effects of berberine in animal and human studies, with a focus on diabetes mellitus.

## 2. Methods

### 2.1. Data Retrieval from the Literature

*In vitro* studies related to berberine and its targets were searched using Pubmed (from inception to April 2012). Search terms used were a combination of “berberine,” “*in vitro,*” “human cell,” and “mechanism.” Only studies using human cell lines were used to extract current berberine targets. Studies using animal cell lines or berberine derivatives or in a language other than English were excluded. Information extracted from these studies includes molecular targets of berberine (name and gene symbols), cell type, effects of berberine, and possible clinical applications.

### 2.2. PANTHER Analysis

Using the PANTHER Classification System, *in vitro* berberine targets were analysed using three approaches: molecular function, biological process, and pathway involvement Table 2. PANTHER is a publicly available database that relates protein sequence evolution to evolution of protein functions and biological roles (http://www.pantherdb.org/).

## 3. Results

### 3.1. Targets of Berberine

A total of 90 berberine targets were identified in our literature search, as shown in Table 1.

Extensive research has been carried out to study the effects of berberine on cancer cells *in vitro. *This may be related to recent discovery of anti-cancer drugs with natural compound origin, for example, paclitaxel and topotecan.

Various human cancer cell lines were used to demonstrate the anti-cancer effects of berberine *in vitro*. These include cancer cell lines of the tongue, stomach, lung, colon, liver, breast, prostate, nasopharyngeal, neurones, epidermal, and blood [18–28]. Berberine has shown to induce cancer cell death via several mechanisms such as regulation of apoptosis proteins and cell cycle arrest.

Berberine treatment increased the expression of apoptotic cell death proteins, promotes cell cycle arrest, and induces cell death in human cancer cell lines. For instance, in human prostate epithelial cells (PWR-1E), berberine-increased expression of BCL2-associated X protein (Bax) was observed after berberine treatment, inducing cell death and demonstrating pro-apoptotic properties [29]. Similar effects of berberine were observed in prostate carcinoma cells (DU145, PC-3, and LNCaP) [21, 30]. Berberine also increased levels of Bax in promyelocytic leukemia cells [31], gastric carcinoma cells [24], and lung cancer cells [20].

Berberine can also promote cell death by the regulation of antiapoptotic proteins. Decreased expression of antiapoptotic Bcl-2 protein was observed in human oral squamous cell carcinoma after berberine treatment [23]. Studies done in other cancer cell lines such as lung cancer, gastric cancer, and prostate cancer also showed reduced levels of Bcl-2 after berberine treatment [20, 21, 24, 30]. Cell cycle arrest at different phases has also been observed in human cancer cell lines after treatment with berberine. In giant cell carcinoma and prostate carcinoma cells, berberine also decreased G_0_/G_1_ phase-associated cyclins (D_1_, D_2_, E, Cdk2, Cdk4, and Cdk6), inducing G_0_/G_1_ arrest and suppressing cell proliferation [21, 25, 30, 32]. Further, in HepG2 cells, berberine acted on B-cell CLL/lymphoma 2 (BCL2), procaspase-3 and -9, and poly (ADP-ribose) polymerase (PARP), induced cell cycle arrest at G_2_/M phase and inhibited cell proliferation [22].

Further, berberine can promote cell death via the regulation of pro- and antiapoptotic proteins. In addition to this, berberine can also promote apoptosis via mitochondrial/caspase pathway. In cancer cell lines (tongue cancer, oral squamous cell carcinoma and prostate epithelial) [18, 23, 29, 33], activation of caspases-3 & -9 promotes G_1_ cell cycle arrest in different human cancer cell lines (lung, stomach, and prostate) [20, 21, 24, 30, 33].

Berberine also showed anti-metastatic properties in several cancer cell lines, acting on 72 kDa type IV collagenase (MMP2), Cdc42 effector protein 1 (CDC42EP1), and ras-related C3 botulinum toxin substrate 1 (RAC1), transforming protein RhoA (RHOA) and urokinase-plasminogen activator A (PLAU) [34, 35]. Further, berberine showed antitopoisomerase I properties [36]; this observation can be useful as topoisomerase I is responsible for DNA replication and antitopoisomerase I compounds can be effective in cancer treatments.

In addition to its effects on cancer cells, berberine also acts on molecular targets related to insulin resistance. In free-fatty-acid-induced insulin resistance muscle cells, berberine improves insulin resistance and improves glucose uptake by decreasing PPAR*γ* and FAT/CD36 protein expression [37]. Another study reported increased insulin receptor (InsR) mRNA and protein expression increases insulin sensitivity in liver cells after berberine treatment [38]. In Caco-2 cells, berberine inhibited alpha-glucosidase and disaccharidases activities, leading to reduced glucose levels [39]. In Hep G_2_ cells, berberine also improved insulin signal transduction through various mechanisms such as decreased phosphorylation of PERK and eLF2-*α*, increased phosphorylation of IRS-1 tyrosine and AKT serine [40]. In intestinal NCI-H716 cells, berberine enhanced glucagon-like peptide 1 (GLP-1) release and promotes proglucagon mRNA expression [41]. These results demonstrate that berberine has great potential for insulin resistance treatment and should be explored further in animal and human studies.

### 3.2. PANTHER Analysis of Berberine Targets

Distribution of berberine therapeutic targets *in vitro* varied in each of these functional classifications. Tables 3, 4, and 5 show various distributions of the most frequent occurring berberine targets *in vitro* based on molecular functions, biological processes, and pathways, respectively.

As shown in Table 3, berberine acts on a diverse range of molecular targets *in vitro*. The most common classes of molecular functions include receptor binding, kinase activity, protein binding, transcription activity, DNA binding, and kinase regulator activity. Known berberine targets *in vitro* from the receptor binding class include epidermal growth factor receptor (EGFR), vascular endothelial growth factor A (VEGFA), interleukin-1*β* (IL1B) and interleukin-6 (IL6), growth/differentiation factor 15 (NAG-1), and glucagon-like peptide (GLP1).

Based on the biological process classification of *in vitro* berberine targets, those targets related to signal transduction, intracellular signalling cascade, cell surface receptor linked signal transduction, cell motion, cell cycle control, immunity system process, and protein metabolic process are most frequently involved (Table 4). *In vitro* berberine targets involved signal transduction include cyclin-dependant kinases (CDK1 and CDK6), inhibitor of nuclear factor kappa-B kinase subunit alpha (CHUK), epidermal growth factor receptor (EFGR), receptor tyrosine-protein kinase (ERBB2), glucagon-like peptide (GCG), growth/differentiation factor 15 (GDF15), interferon beta (IFNB1), interleukins (IL1B, IL2RA, and IL6), potassium voltage-gated channel subfamily H member 2 (KCNH1), mitogen-activated protein kinases (ERK1, ERK2, and MAPK8), nuclear factor-kappa-B p50 subunit (NFKB1), NF-kappa-B inhibitor alpha (NFKB1A), urokinase-plasminogen activator (PLAU), Ras-related C3 botulinum toxin substrate 1 (RAC1), Rho-associated protein kinase 4 (RHO), transforming protein RhoA (RHOA), proto-oncogene tyrosine-protein kinase ROS (ROS1), vascular endothelial growth factor A (VEGFA).

According to the PANTHER Classification System, *in vitro* berberine targets correlate with a mixture of biological pathways, such as Alzheimer disease-presenilin and secretase pathways, angiogenesis, apoptosis signalling pathway, FAS signalling pathway, Huntington disease, inflammation mediated by chemokine and cytokine signalling pathways, interleukin signalling pathway, and p53 pathways (Table 5).

The targets of berberine distributed across a large number of PANTHER classifications of molecular functions, biological processes, and pathways. This can be an advantage in terms of drug discovery using berberine. Seen that berberine targets are involved in a wide range of molecular activities, in turn, can alter many pathological states of the body. Thus, berberine can be explored for the treatment of different diseases. On the other hand, the nature of multitargeting of berberine lacks in target specificity which can become difficult for drug design. Further, because berberine can have interactions with so many molecular structures and involve in different pathways, much attention must be paid to avoid interactions with other therapeutic drugs.

### 3.3. Data from *In Vivo* Studies with a Focus on Diabetes Mellitus

In China, Huang Lian (*Rhizoma coptidis*) has been used to treat diabetes for more than 1,400 years [16]. Berberine is one of the main active alkaloids present in *Rhizoma Coptidis* and has shown to have good hypoglycaemic effects *in vitro* [37–39, 42]. Further, the chemical structure of berberine is different from the commonly used other hypoglycaemic agents such as sulphonylureas, biguanides, thiazolidinediones, or acarbose [14]. Thus, it is meaningful to investigate the efficacy and safety of berberine treatments for diabetes mellitus to confirm the possibility of berberine serving as a new class of antidiabetic medications. Extensive research has been done to investigate the hypoglycaemic effects of berberine in animal models. This section will highlight the effects of berberine in diabetic animal studies, focusing on different mechanisms of actions of berberine.

Hyperglycemia is a hallmark metabolic abnormality associated with metabolic diseases such as type 2 diabetes. Berberine has shown to significantly decrease fasting blood glucose levels in diabetic rats (diet or drug induced), this has been observed in a number of studies [43–46]. Berberine can reduce fasting blood glucose level via different mechanisms. For example, Liu et al. [43] reported that berberine reduced fasting blood glucose (FBG) levels by inhibiting intestinal disaccharidases in a concentration-dependent manner. Xia et al. [46] reported berberine reduced fasting glucose level via the inhibition of gluconeogenesis, via decreased *PEPCK* and *G6Pase* genes in the liver, reduced hepatic steatosis, and inhibition of FAS expression.

Current diabetes therapies do not address the key driver of this condition, *β*-cell dysfunction [47, 48], and do not alter the progressive nature of insulin secretory deficit [49]. Berberine increased pancreatic *β*-cell numbers and *β*-cell mass in streptozotocin-induced diabetic rats [41, 50]. It also reversed pathological changes of pancreatic *β*-cells in diabetic rats induced by streptozotocin and diet [51]. Further, in berberine treated diabetic rats, the pancreatic and plasma insulin levels increased after glucose load, reducing blood glucose levels [41, 50]. These observations are significant as berberine may be explored further as an additional therapy to existing antidiabetic drugs to effectively preserve *β*-cell functions, reverse *β*-cell damage, and promote insulin secretion in diabetes patients.

Further to *β*-cell dysfunction and insulin secretory deficit in diabetes, defects in insulin receptor (InsR) expression or function can cause insulin resistance and diabetes mellitus [52]. Thus, regulation of InsR expression may improve insulin resistance in diabetes mellitus. Berberine increases InsR mRNA and protein expression in human liver cells and in animal model in a dose- and time-dependent manner [38]. Berberine upregulates InsR and leads to enhanced insulin signalling pathway, confirming berberine as an insulin sensitiser.

Glucagon-like peptide 1 (GLP-1) is an intestinal peptide hormone released in response to food ingestion [53]. GLP-1 enhances meal-related insulin secretion and promotes glucose tolerance. In streptozotocin-induced rats, berberine enhanced GLP-1 release and promotes proglucagon mRNA expression, increased beta cell mass and pancreas insulin levels after glucose load [41]. This observation was in line with the groups, previous experiments *in vitro*. Lu et al. [50] also reported that berberine increased proglucagon mRNA expression and plasma insulin levels in streptozotocin-induced diabetic rats. The glucagon gene encodes GLP-1 and the increased expression of proglucagon mRNA assists in controlling the blood glucose homeostasis.

Berberine also reduced body weight and caused a significant improvement in glucose tolerance without altering food intake in *db/db* mice [54]. Oral glucose tolerance improvement in diabetic rats after berberine treatment has also been observed in other studies [55, 56].

Long-term hyperglycaemia can lead to increased risk of cardiovascular complications. In hyperglycemia and hypercholesterolemia rats with injured cardiac functions, berberine (15, 30 mg/kg/day, i.g for 6 weeks) increased cardiac output, left ventricular systolic pressure, and +*dp*/*dt*_max⁡_ by 64, 16, and 79%, but decreased left ventricular end diastolic pressure and −*dp*/*dt*_max⁡_ by 121 and 61% in the rats receiving HSFD/streptozotocin, respectively, when compared with the untreated rats of hyperglycemia and hypercholesterolemia [57]. Berberine caused significant increase in cardiac fatty acid transport protein-1 (159%), fatty acid transport proteins (56%), fatty acid beta-oxidase (52%), and glucose transporter-4. These results demonstrate the cardioprotective functions of berberine in hyperglycemia/hypercholesterolemia through alleviating cardiac lipid accumulation and promoting glucose transport 4 [57]. Another study also showed improved vasorelaxation in impaired aorta in diabetic rats after berberine treatment (100 mg/kg/day, 8 weeks) [45]. Thus, in addition to its hypoglycaemic effects, berberine can also be investigated for cardiomyopathy in diabetes.

Berberine also regulates lipid metabolism which is closely related to diabetes. In rats with induced diabetic hyperlipidemia, berberine (75, 150, 300 mg/kg/day for 16 weeks) effectively reduced liver weight and liver/body weight ratio, levels of total cholesterol, triglycerides, and low-density lipoprotein-cholesterol [58]. In rats with a high fat diet, berberine significantly reduced body weight, alleviated liver steatosis, and improved insulin resistance [59]. This observation indicates that berberine can be an effective treatment for diabetes with obesity.

Clinically, preeminent factors for monitoring glycaemia and evaluating the risks of complications of diabetes include FBG, haemoglobin A_1c_ (HbA_1c_) [60]. Triglyceride synthesis is closely associated with glucose metabolism so serum triglyceride levels are determined. Clinical studies often measure FBG, HbA_1c_, and triglyceride levels, along with other factors to study the hypoglycaemic effects of berberine. The efficacies of berberine in type 2 diabetes patients have been reported. Through literature search, key clinical studies on berberine effects on type 2 diabetes patients are summarised.

Zhang et al. [61] conducted a randomized, double-blind, placebo-controlled multicenter trial (*n* = 116). The authors found that when berberine (1.0 g daily) was administered for 3 months in type 2 diabetes patients with dyslipidemia, the fasting and postload plasma glucose levels decreased from 7.0 ± 0.8 to 5.6 ± 0.9 and from 12.0 ± 2.7 to 8.9 ± 2.8 mM/L, HbA_1c_ from 7.5 ± 1.0% to 6.6 ± 0.7%. Further, in the treatment group, triglyceride levels were reduced from 2.51 ± 2.04 to 1.61 ± 1.0 mM/L, total cholesterol from 5.31 ± 0.98 to 4.35 ± 0.96 mM/L, and LDL-cholesterol from 3.23 ± 0.81 to 2.55 ± 0.77 mM/L. Results from the treatment group was significant compared to the control group. In the treatment group, patient's body weight was also significantly reduced. Mild-to-moderate constipation was reported in 5 patients from the treatment group and 1 patient from the control group; however, this finding was not statistically significant. No other adverse events were reported. At 3 months, berberine was found to be effective in lowering blood glucose, lipids, body weight, and blood pressure with a good safety profile.

Yin et al. reported a 3-month study comparing berberine to antidiabetic drug metformin (0.5 g t.i.d) [14]. In this study, berberine exhibited identical effect as metformin in the regulation of glucose metabolism, significant decreases in HbA_1c_ (by 2%, *P* < 0.01), FBG (by 3.8 mmol/L; *P* < 0.01), and postprandial blood glucose (PBG) (by 8.8 mmol/L; *P* < 0.01). Further, the regulation of lipid metabolism was better in the berberine group than the metformin group. Triglycerides and total cholesterol levels were significantly lower than in the metformin group (*P* < 0.05). At the same time, the same group of researchers used berberine as a combination therapy to evaluate its additive or synergistic effects on the commonly used hypoglycemic agents, such as sulphonylureas, biguanides, thiazolidinediones, and acarbose. Patients were given 500 mg berberine three times daily for 3 months in addition to their previous treatment. At week 5, berberine significantly (*P* < 0.01) reduced HbA_1c_ (from 8.1% to 7.3%), FBG, PBG, and fasting insulin levels. Blood lipids including triglyceride, total cholesterol, and LDL-C decreased significantly lowered compared to baseline. In both studies, incidences of gastrointestinal adverse events were observed, including diarrhea, constipation, flatulence, and abdominal pain. Interestingly, patients did not suffer from severe gastrointestinal adverse events when berberine was used alone and in combination therapy; adverse effects disappeared after berberine dosage was reduced. No pronounced elevation in liver enzymes or creatinine was observed, suggesting that berberine did not cause damage to the liver or kidneys.

Another clinical study [62] randomly divided 97 type 2 diabetes mellitus patients into berberine treatment (1 g/day) for 2 months, using metformin therapy (1.5 g/day) and rosiglitazone group (4 mg/b.i.d) as reference groups. Blood samples were taking before and after treatments to measure FBG, HbA_1c_, triglyceride, and serum insulin levels. Compared to values prior to treatment, berberine significantly lowered FBG by 25.9% (*P* < 0.001), HbA_1c_ by 18.1% (*P* < 0.00), and triglycerides by 17.6% (*P* < 0.01). The hypoglycaemic effects of berberine were comparable to metformin and rosiglitazone. Serum insulin level was declined significantly (*P* < 0.01) by 28.2%; this indicates increased insulin sensitivity in peripheral tissues by berberine treatment. Peripheral blood lymphocytes from berberine treated patients were isolated to examine the InsR expression. The surface expression of InsR significantly elevated by 3.6-fold after berberine treatment.

Metformin and rosiglitazone are not recommended for use in diabetic patients with liver function damage [54, 63]. So the effect of berberine was tested in hyperglycaemic patients with hepatitis. Hepatitis B and C patients with hyperglycaemia received berberine at 1 g/day for 2 months. In both diabetic hepatitis B and C patients, berberine significantly reduced FBG and triglyceride levels. Berberine treatment also reduced the elevated alanine transaminase and aspartate aminotransferase levels in these patients. Overall, berberine is safe and effective in hyperglycaemic patients with liver function damage.

Table 6 compares clinical studies of berberine in diabetes patients. Across the studies, berberine has shown to significantly reduce FBG, PBG, and HbA_1c_ levels. Berberine also demonstrated ability to reduce triglyceride and cholesterol levels. Minimal gastrointestinal side effects were shown but no liver or kidney damage was observed. These observations in diabetes patients demonstrate that berberine is a safe and effective antidiabetic agent.

## 4. Discussion

The “rule-of-five” analysis by Lipinski et al. [7] shows that poor absorption or permeation of a compound is more likely when there are more than five hydrogen-bond donors; the molecular mass is more than 500 Da; the lipophilicity is high (expressed as  *c*Log *P* > 5); the sum of nitrogen and oxygen is more than 10. Specific structural and physiochemical properties, such as “rule-of-five,” are required for clinical drugs to have sufficient levels of efficacy, bioavailability, and safety, which define target sites to which drug-like molecules can bind [4].

Plant compounds exhibit enormous structural diversity and only a small portion of the diversity has been explored for its pharmacological potential [64]. In recent years, herbal compounds have been source of new drugs [64]. Approximately 28% of new molecular entities (NMEs) between 1981 and 2002 were natural products or natural product derived; further to this, 20% of these NMEs were natural product mimics [65]. There are a number of successful plant-derived drugs, especially in anti-cancer treatment. Medicinal herbal compounds have become an important source for the discovery of new drugs. Further, drugs derived from medicinal plants can also be used as drug leads suitable for optimization by medicinal and synthetic chemists [65].

As Chinese herbal medicine becomes increasingly popular in the west, researchers are spending more time looking into mechanisms of actions of crude extracts and herbal compounds such as berberine. In recent years, extensive research has been done to explore the effects of berberine on various cell lines *in vitro*. In cell-based studies, berberine has shown effects on multiple molecular targets and alters various biological pathways. Berberine associates with a range of conditions, particularly diabetes, hyperlipidemia, and cancer. Many *in vitro* studies showed potent anticancer properties of berberine against various cancer cells. This observation is valuable in the search for new anti-cancer therapeutics with potent anti-cancer effects but reduced side effect. So berberine may potentially be developed into an anticancer agent, like other natural compounds (taxol, camptothecin) that have been developed and used as anticancer agents.

Diabetes mellitus is a major health problem around the world and its prevalence is on the rise. Diabetes mellitus drug therapy is limited by availability of effective medications, as existing oral hypoglycaemic agents often have side effects and fails in long-term administration [14]. Berberine has shown significant results in fasting blood glucose levels reduction, increase in insulin sensitivity, and improvement in insulin resistance *in vitro*, in diabetic animal models and in diabetic patients. Further, berberine shows mechanism that current antidiabetic drugs do not have. For instance, berberine has shown effects on pancreatic *β*-cell number and mass improvement [41, 50, 51]. In addition, berberine has a good safety profile and does not show side effects such as hypoglycaemia, weight gain, or liver and kidney damage. Metformin and rosiglitazone are not recommended for use in diabetic patients with liver function damage [54, 63]. Berberine has shown to be effective in the reduction of blood glucose level and is safe in diabetic patients with viral hepatitis [62]. Berberine can therefore be investigated as an effective diabetes therapy with patients with liver function damage. In addition to its hypoglycemic effects in diabetic patients, berberine also reduced triglyceride and cholesterol levels. Abnormalities in lipid metabolism often deteriorate diabetes and cause complications. The regulation of lipid metabolism in diabetes patients by berberine may have clinical significance in managing diabetic patients with hyperlipidemia. Although there are only a small number of clinical studies and evidence is limited, current reports still show a promising future for berberine being developed into a new antidiabetic agent.

In China, berberine has been manufactured into the over-the-counter drug Huang Lian Su Pian, also known as Coptis Extract Tablets for the treatment of traveler's diarrhea [14, 17]. However, *in vitro* and *in vivo* studies have shown that berberine has potent anti-cancer, antidiabetic, antilipidemic, and anti-inflammatory effects. Therefore, further clinical studies are warranted to investigate the potential of berberine in the application of cancer and diabetes treatments in the future.

Pharmacological activity of CHMs begins with the binding of the active components to their molecular targets. CHMs are considered as typical multitherapeutics that can interact simultaneously with multiple targets. The origins and the progression of diseases are multifactorial. Complex disorders such as cancer, cardiovascular disease, and depression tend to result from multiple molecular abnormalities, not from a single defect [66]. Biochemical and genetic studies revealed the molecular mechanism that underlie common illnesses [66]. Reports show that targets for neoplasm diseases, circulatory system diseases, infectious diseases, and nervous system and sense organs disorders constitute the largest number of targets [1]. Because drug targets are presented at the molecular level, increased knowledge of herbal targets can facilitate deeper understanding of complex diseases at its fundamental level. In turn, it is likely to determine the optimal molecular targets for therapeutic intervention [6].

Further to assisting the molecular dissection of the mechanism of action of CHMs, knowledge on herbal targets makes it possible to use disease specific targets and design more desirable herbal drugs/formulas with increased specificity and efficacy. Target-oriented synthesis in drug discovery involves in preselected protein targets [67]. Binding of drugs to preselected protein target/s is dependent on which biological pathway the drug is aimed to modulate the target or the diseased pathway(s) [67]. Target and disease specific drug design results in improved efficacy and reduced side effects, especially in high impact diseases that require more effective and more treatment options. However, due to the fact that diseases often involve in multiple molecular abnormalities, diversity-oriented syntheses are used in efforts to identify simultaneously therapeutic protein targets and their small-molecule regulators [67]. Target-oriented drug design allows more focused drug design, which in turn costs less time and money for pharmaceutical companies.

Protein structure of well-validated old and new targets should be able to guide the chemical effort directed at new drugs [68]. Study of various aspects of known targets including molecular mechanism of their binding agents and related adverse effects is useful for finding clues to new target identification [9]. Based on the knowledge of molecular targets and molecular understanding of disease state and using this knowledge will allow some direction in identifying potential targets. Potential herbal targets may come from the same class as confirmed therapeutic targets and have similar physiological functions, or maybe a structure along a biological pathway. Additionally, with increased number of potential targets from ~500 to >5,000, the nature of pharmaceutical research has changed. This increase in numbers has given researchers more opportunities to discover and design new and improved drugs.

Target selection may be one of the most important determinants of attrition and the overall R&D productivity. There are few ways to overcome this challenge and improve the target selection process, in turn, improving R&D productivity. First of all, researchers can discover new target classes. Targets of herbal medicine are becoming a popular resource to find new target classes. In addition, increased understanding of genetic variations/polymorphisms of drug targets or drug metabolising enzymes can assist in target selection and drug metabolism. Further, the use of new technology can help to speed up the early exploratory discovery phase of drug discovery.

In summary, updated knowledge of herbal targets is valuable contribution to complex disease understanding and clinical responses. Further, drug discovery and development from herbal medicines can be supported by new target discovery and target-focused drug design. This will speed up the exploratory phase of drug R&D and benefit the pharmaceutical industry in terms of cost and time.

## Figures and Tables

**Table 1:** Potential targets of berberine.

Target names	Target gene symbol	Cells	Effects	Possible clinical applications	References
72 kDa type IV collagenase	*MMP2*	HUVECs, tongue cancer SCC-4 cells, gastric carcinoma SNU-5 cells, lung cancer A549 cells, and U-87 glioma cells	Downregulation of MMP2 mRNA and protein expression, reduced MMP-2 levels	Antimetastatic	[18, 69–72]

Acetyl-Coenzyme A carboxylase-*α*	*ACACA*	HepG2 hepatoma cells	Phosphorylation	Antihyperlipidemic	[73]

*α*-Fetoprotein	*AFP*	HepG2 hepatoma cells	Reduced secretion of alpha fetoprotein	Apoptosis	[74]

Amyloid-*β* (A4) precursor protein (peptidase nexin-II, Alzheimer disease)	*APP*	Neuroglioma H4 cells	Reduces amyloid-*β* peptide (A*β*) levels via modulation of APP	Alzheimer disease	[75]

Bcl-X	*BCL2L1*	Colonic carcinoma cells, HepG2 cells/A549 cells, prostate carcinoma cells (DU145 and PC-3, LNCaP), Jurkat cells	JNK/p38 pathway and induction of ROS production. Decreased expression	Cell apoptosis, anticancer, and anti-inflammatory	[19–21, 30, 76, 77]

Arylamine *N*-acetyltransferase 1	*NAT*	Leukemia HL-60 cells, colon tumour cells, brain tumour cells (G95/VGH and GBM 8401)	Decrease in *N*-acetyltransferase (NAT) protein and expression of mRNA	Anticancer (leukemia, colon cancer, brain tumour, etc.)	[78–80]

ATP-binding cassette subfamily G member 2	*ABCG2*	MCF-7 breast cancer cells	Decrease in ABCG2 expression	Breast cancer	[81]

Baculoviral IAP repeat-containing protein 2 (antiapoptosis factor c-IPA-1)	*BIRC2*	Jurkat cells, colonic carcinoma cells (SW620)	Suppresses expression of antiapoptosis factor IAP1	Anticancer	[19, 77]

Baculoviral IAP repeat-containing protein 3	*BIRC3*	Jurkat cells	Suppresses expression of antiapoptosis factor IAP2	Anticancer	[77]

Baculoviral IAP repeat-containing protein 5 (Survivin)	*BIRC5*	Jurkat cells	Suppresses expression of survivin	Anticancer and anti-inflammatory agent	[77]

B-cell CLL/lymphoma 2	*BCL2*	HepG2 cells, oral squamous cell carcinoma, tongue cancer SCC-4 cells, colonic carcinoma cells, lung cancer cells, breast cancer MCF-7 (estrogen receptor+) cells, prostate carcinoma cells (DU145 and PC-3, LNCaP), activated rheumatoid arthritis fibroblast-like synoviocytes (RAFLSs)	Bcl-2 Downregulation	Cell apoptosis, cancer, and ER antagonist adjuvant therapy	[19–23, 30, 34, 82, 83]

B-cell lymphoma 3-encoded protein	*Bcl-3*	Gastric carcinoma SNU-5 cells	Downregulation of Bcl-3	Gastric cancer	[24]

Bcl2 antagonist of cell death	*BAD*	Human oral squamous cell carcinoma	Increased expression of proapoptotic BAD protein	Antitumour	[23]

BCL2-associated X protein	*BAX*	Gastric carcinoma SNU-5 cells, prostate carcinoma cells (DU145, PC-3, LNCaP and PWR-1E), leukemia HL-60, tongue cancer SCC-4 cells, lung cancer cells, activated rheumatoid arthritis fibroblast-like synoviocytes (RAFLSs)	Upregulation of Bax, increased expression. G2/M phase arrest	Cell apoptosis, gastric cancer	[18, 20, 21, 24, 29, 30, 83, 84]

BH3-interacting domain death agonist p11	*BID*	Colonic carcinoma cells/HepG2 cells	JNK/p38 pathway and induction of ROS production	Induction of apoptosis	[19, 76]

C/EBP homologous protein (CHOP) or growth arrest- and DNA damage-inducible gene 153 (GADD153) or DNA damage-inducible transcript 3	*GADD153/DDIT3*	Cervical cancer Ca Ski cells	Induced expression of GADD153	Cervical cancer	[85]

CASP8 and FADD-like apoptosis regulator subunit p12	*CFLAR/cFLIP*	Jurkat cells	Suppresses expression of cFLIP	Anticancer and anti-inflammatory	[77]

Caspase 3	*CASP3*	Tongue cancer SCC-4 cells, neuroblastoma (SK-N-SH), glioblastoma T98G cells, gastric carcinoma SNU-5 cells, HL-60 cells, prostate carcinoma cells (DU145, PWR-1E, PC-3 and LNCaP), colonic carcinoma cells, hepatoma cells, oral squamous cell carcinoma, promonocytic U937 cells, lung cancer A549, H1301 cells, activated rheumatoid arthritis fibroblast-like synoviocytes (RAFLSs), BIU-87 and T24 bladder cancer cells	Activation of caspase-3, G2/M phase arrest	Anticancer	[18–21, 23–25, 30, 33, 76, 83, 86–89]

Caspase 8	*CASP8*	Tongue cancer SCC-4 cells, colonic carcinoma cells, hepatoma cells, oral squamous cell carcinoma	Activated caspase 8	Anticancer	[18, 19, 23, 76]

Caspase 9	*CASP9*	Tongue cancer SCC-4 cells, glioblastoma T98G, oral squamous carcinoma, promonocytic U937 cells, prostate carcinoma cells (DU145 and PC-3, LNCaP), activated rheumatoid arthritis fibroblast-like synoviocytes (RAFLSs), BIU-87 and T24 bladder cancer cells	Activation of caspase 9	Cell apoptosis, anticancer	[18, 21, 23, 30, 33, 83, 86, 87, 89, 90]

Cdc42 effector protein 1	*CDC42EP1*	Nasopharyngeal carcinoma (HONE1) cells	Suppression of Rho GTPases activation (Cdc42)	Cancer metastasis inhibition	[91]

Cell division protein kinase 6	*CDK6*	Prostate carcinoma cells (DU145 and PC-3, LNCaP), activated rheumatoid arthritis fibroblast-like synoviocytes (RAFLSs)	Decrease in Cdk6	Cell apoptosis, cancer	[21, 30, 83]

Cellular tumor antigen p53	*TP53*	Gastric carcinoma SNU-5 cells, osteosarcoma	Increased expression of p53 protein, cell cycle arrest at G1G2/M phase arrest	Anticancer (gastric cancer, osteosarcoma)	[24, 92]

Chemokine (C-C motif) ligand 2 (monocyte chemotactic protein-1)	*CCL2*	Retinal pigment epithelial cell line	Inhibits CCL2 (MCP-1) expression	Anti-inflammatory	[93]

Cyclic AMP-dependent transcription factor ATF-3	*ATF3*	Colorectal cancer cells	Induces ATF3 expression	Colorectal cancer	[94]

Cyclin-dependant kinase 1/cell division control protein 2 homolog	*CDK1/CDC2*	HL-60 cell, gastric carcinoma SNU-5 cells	Inactivation of Cdc2 (CDK1) or decreased protein expression	Antiproliferative and proapoptotic	[24, 95]

Cyclin E1	*CCNE1*	Neuroblastoma (SK-N-SK), glioblastoma T98G cells, activated rheumatoid arthritis fibroblast-like synoviocytes (RAFLSs)	Decrease in cyclin E	Anticancer	[25, 83, 90]

Cyclin-dependent kinase 2	*CDK2*	Neuroblastoma (SK-N-SK), glioblastoma T98G cells, prostate carcinoma cells (DU145 and PC-3, LNCaP), activated rheumatoid arthritis fibroblast-like synoviocytes (RAFLSs)	Decrease in Cdk2	Cell apoptosis, anticancer	[21, 25, 30, 83, 90]

Cyclin-dependent kinase 4	*CDK4*	Neuroblastoma (SK-N-SK), glioblastoma T98G cells, prostate carcinoma cells (DU145 and PC-3, LNCaP), activated rheumatoid arthritis fibroblast-like synoviocytes (RAFLSs)	Decrease in Cdk4	Cell apoptosis, anticancer	[21, 25, 30, 83, 90]

Cyclin-dependent kinase inhibitor 1 (p21)	*CDKN1A*	Brest cancer MCF-7 (estrogen receptor+) cells, epidermoid carcinoma A431 cells, activated rheumatoid arthritis fibroblast-like synoviocytes (RAFLSs)	Increased expression of p21	Breast cancer, ER antagonist adjuvant therapy	[21, 30, 82, 83]

Cyclin-dependent kinase inhibitor 1B (P27/KIP1)	*CDKN1B*	Epidermoid carcinoma A431 cells, activated rheumatoid arthritis fibroblast-like synoviocytes (RAFLSs)	Increased expression of Cdki proteins	Cell apoptosis, cancer	[21, 30, 83]

Cytochrome c-1	*CYC1*	Tongue cancer SCC-4 cells, colonic carcinoma cells, promyelocytic leukemia HL-60 cells	Release of cytochrome c-1	Anticancer	[18, 19, 84, 86]

CYP2C9	*CYP2C9*	Recombinant CYP	Inhibition of CYP2C9	Drug interactions	[96]

CYP2D6	*CYP2D6*	Recombinant CYP	Inhibition of CYP2D6	Drug interactions	[96]

CYP3A4	*CYP3A4*	Caco-2 cells, patients	CYP3A4 Downregulation and inhibition	Drug interactions	[97, 98]

Dipeptidyl-peptidase 4 (CD26, adenosine deaminase complexing protein 2)	*DPP4*	Recombinant DPP4	Inhibition of DPP4	—	[99]

Early activation antigen CD69	*CD69*	Human peripheral lymphocytes	Reduced expression of CD69	Immunosuppressive agent	[100]

Epidermal growth factor receptor	*EGFR*	Brest cancer MCF-7 (estrogen receptor+) cells	EGFRdownregulated	Breast cancer, ER antagonist adjuvant therapy	[82]

Ezrin	*EZR*	Nasopharyngeal carcinoma 5–8F cells	Ezrin inhibition	Anticancer	[26]

G1/S-specific cyclin-D1	*CCND1*	Giant cell carcinoma cell line, HL-60 cell, prostate carcinoma cells (DU145 and PC-3, LNCaP), Jurkat cells, neuroblastoma (SK-N-SK), activated rheumatoid arthritis fibroblast-like synoviocytes (RAFLSs)	Inhibits expression of cyclin D1	Antiproliferative and proapoptotic, anticancer, anti-inflammatory	[21, 25, 30, 32, 77, 83, 95]

G1/S-specific cyclin-D2	*CCND2*	Prostate carcinoma cells (DU145 and PC-3, LNCaP), activated rheumatoid arthritis fibroblast-like synoviocytes (RAFLSs)	Decrease in cyclin D2	Cell apoptosis, cancer	[21, 30, 83]

G1/S-specific cyclin-E1	*CCNE1*	Prostate carcinoma cells (DU145 and PC-3, LNCaP), activated rheumatoid arthritis fibroblast-like synoviocytes (RAFLSs)	Decrease in cyclin E	Cell apoptosis, cancer	[21, 30, 83]

G2/mitotic-specific cyclin-B1	*CCNB1*	Gastric carcinoma SNU-5 cells	Decreased cyclin B, G2/M phase arrest	Cell apoptosis, anticancer	[24]

Glucagon-like peptide (GCG/GLP-1/GLP-2)	*GCG*	NCI-H716 cells	Enhanced glucagon-like peptide (GLP)-1	Antidiabetic agent	[42]

Growth/differentiation factor 15 (NAG-1)	*GDF15*	Colorectal cancer cells	Induces NAG-1 expression	Colorectal cancer	[94]

Hypoxia-inducible factor 1*α*	*HIF1A*	HUVECs, HepG2 cells	Prevention and reduction of HIF-1 alpha expression	Tumour angiogenesis	[101, 102]

Induced myeloid leukemia cell differentiation protein Mcl-1	*MCL1*	Oral cancer cells	Inhibition of Mcl-1 expression	Induced apoptosis	[103]

Inhibitor of NF-*κ*B kinase subunit alpha (I*κ*B kinase)	*CHUK(IKK)*	Jurkat cells	Inhibition of I*κ*B kinase (IKK)	Anticancer and anti-inflammatory agent	[77]

Interferon-*γ*	*IFNB1*	Brest cancer MCF-7 (estrogen receptor+) cells	IFN-beta upregulated	Breast cancer, ER antagonist adjuvant therapy	[82]

Interleukin 8	*IL8*	Retinal pigment epithelial cell line	Inhibits IL-8 expression	Anti-inflammatory	[93]

Interleukin-1*β*	*IL1B*	Fibroblasts (HFL1)	Induces IL-1B productions	Pulmonary inflammation	[104]

Interleukin-2 receptor *α*-chain	*IL2RA/CD25*	Human peripheral lymphocytes	Reduced expression of CD25	Immunosuppressive agent	[100]

Interleukin-6	*IL6*	Keratinocytes	Reduces and IL-6 expression	Antiskin ageing agent	[105]

Low-density lipoprotein receptor (familial hypercholesterolemia)	*LDLR*	HepG2 cells	Increased mRNA and protein expression	Hyperlipidemia	[106–108]

Matrix metallopeptidase 1 (27 kDa interstitial collagenase)	*MMP1*	Dermal fibroblasts, U-87 glioma cells	MMP-1 expression decreased	Antiskin ageing agent, anticancer	[70, 109]

Matrix metallopeptidase 9 (gelatinase B, 92 kDa gelatinase, 92 kDa type IV collagenase)	*MMP9*	Tongue cancer SCC-4 cells, keratinocytes, gastric carcinoma SNU-5	Inhibition	Anticancer	[34, 70, 105]

Matrix metalloproteinase-16	*MMP16*	Jurkat cells	Suppresses expression of MMP-16	Anticancer and anti-inflammatory agent	[77]

Mitogen-activated protein kinase 3	*ERK1/MAPK3*	Peripheral blood monocytes (PBMC)	ERK1 protein expression inhibition	Antiatherosclerotic effects	[110]

Mitogen-activated protein kinase 4	*ERK2/MAPK4*	Peripheral blood monocytes (PBMC)	ERK2 protein expression inhibition	Antiatherosclerotic effects	[110]

Mitogen-activated protein kinase 8 (JNK)	*MAPK8*	Peripheral blood monocytes (PBMC)	Jun N-terminal kinase (JNK) protein expression inhibited at high levels of BBR	Antiatherosclerotic effects	[19, 110]

M-phase inducer phosphatase 1	*CDC25A*	HL-60 cell	Phosphorylation and degradation of Cdc25A	Antiproliferative and proapoptotic	[95]

Multidrug resistance protein 1 (P-gp, P-gp-170)	*ABCB1*	Tumour cell lines	Significant inhibited P-gp multidrug resistance (MDR) activity	MDR activity reversal	[111]

		Hepatoma HepG2 cells	Upregulated multidrug resistance transporter (P-gp-170) expression	Reduced retention of chemotherapeutic agents	[112]

Myc proto-oncogene protein	*MYC*	U-87 glioma cells	Myc level decreased	Malignant glioma and cancer development	[71]

NF-*κ*B inhibitor-*α*	*NFKBIA*	Lung epithelial cells (A-549)	Inhibition of *κ*B-*α* phosphorylation and degradation	Pulmonary inflammation	[104]

Nuclear factor NF-*κ*B p50 subunit (NF-*κ*B)	*NFKB1*	Jurkat cells, osteoblastic cells, HepG2 cells	Inhibit NF-*κ*B production and suppress NF-*κ*B	Anticancer and anti-inflammatory agent, alcohol liver disease, osteoclast formation	[77, 113–115]

Nuclear receptor subfamily 3, group C, member 1 (glucocorticoid receptor)	*NR3C1*	HepG2 cells	Reduced GR levels	Cell growth arrest	[74]

Nucleophosmin (nucleolar phosphoprotein B23) and telomerase	*NPM1*	Leukemia HL-60 cells	Downregulation of nucleophosmin/B23 and telomerase activity	Cancer	[116]

Peroxisome proliferator-activated receptor-*γ*	*PPARG*	Free-fatty-acid-induced insulin resistance muscle cells-L6 myotubes, 3T3-L1 preadipocytes	Decreased expression	Antidiabetic	[37, 117]

Platelet glycoprotein 4	*CD36/FAT*	Free-fatty-acid-induced insulin resistance muscle cells-L6 myotubes	Decreased expression	Antidiabetic	[37]

Poly (ADP-ribose) polymerase family, member 1	*PARP*	HepG2 cells/hepatoma cells, colonic carcinoma cells, prostate cancer cells (PC-3), prostate carcinoma cells (DU145 and PC-3, LNCaP), activated rheumatoid arthritis fibroblast-like synoviocytes (RAFLSs)	Cleavage of poly (ADP-ribose) polymerase. Activation of PARP	Cell apoptosis, Anticancer	[19, 21, 22, 30, 76, 83, 87]

Potassium voltage-gated channel subfamily H member 2	*KCNH2/HERG1*	Leukemic stem cells (LSCs)	Inhibits HERG1 K (+) channels of leukemic cells	Inhibits AML cell migration	[35]

Processed sterol regulatory element-binding protein 2	*SREBP2*	HepG2 cells	Reduction of SREBP2	Hyperlipidemia	[101]

Proprotein convertase subtilisin/kexin type 9	*PCSK9*	HepG2 cells	Suppression of PCSK9 mRNA and protein levels	Hyperlipidemia	[101, 107]

Prostaglandin G/H synthase 2	*PTGS2/COX2*	Peripheral blood monocytes (PBMC), oral cancer cell lines OC2 and KB cells, breast cancer MCF-7 (estrogen receptor+) cells, Jurkat cells, colon cancer cells	Decrease of Cox-2 mRNA and protein expression	Antiatherosclerotic effects, anti-inflammatory, anticancer, breast cancer ER antagonist adjuvant therapy, Anticancer	[27, 77, 82, 103, 110, 118]

Proto-oncogene tyrosine-protein kinase ROS	*ROS1*	HUVECs	Inhibition of ROS generation	Protects LDL oxidation and prevents ox-LDL-induced cellular dysfunction	[19, 119]

Ras-related C3 botulinum toxin substrate 1	*RAC1*	Nasopharyngeal carcinoma (HONE1) cells	Suppression of Rho GTPases activation (Rac1)	Cancer metastasis inhibition	[91]

Receptor tyrosine-protein kinase erbB-2	*ERBB2/HER2*	Brest cancer MCF-7 (estrogen receptor+) cells	HER2 downregulated	Breast cancer, ER antagonist adjuvant therapy	[82]

Rho-associated protein kinase 1	*ROCK1/RHO*	Nasopharyngeal carcinoma 5–8F cells	Suppression of Rho kinase activity	Anticancer	[91]

Runt-related transcription factor 2	*RUNX2*	Osteoblast cells	Promotes transcriptional activity of Runx2	Osteoblast differentiation and bone formation in osteoporosis	[120]

SDF-1-*α* (3–67) (SDF-1)	*CXCL12*	Acute myeloid leukemia (AML)	Reduces SDF-1 chemokine	Inhibits AML cell migration	[35]

Sucrase-isomaltase (*α*-glucosidase)	*SI*	Caco-2 cells	Inhibit alpha-glucosidase	Antihyperglycaemic	[39]

Topoisomerase (DNA) I	*Top1*	Recombinant human topoisomerase I	Top1 inhibition	Anticancer	[121]

Transcription factor AP-1	*AP-1*	Hepatoma cells, MDA-MB-231 breast cancer cells, giant cell carcinoma cell line, colon cancer cells, U-87 glioma cells, HeLa cells	Inhibition of AP-1 activity, AP-1 DNA suppression	Antitumor activity, Anticancer	[27, 32, 71, 115, 118, 122–124]

Transforming protein RhoA	*RHOA*	Nasopharyngeal carcinoma (HONE1) cells	Suppression of Rho GTPases activation (RhoA)	Cancer metastasis inhibition	[91]

Tumor necrosis factor-*α*	*TNFA*	Macrophages, fibroblasts (HFL1)	Inhibition of TNF-*α*	Anti-inflammatory	[104, 125]

Urokinase-plasminogen activator	*PLAU*	Lung cancer A549 cells, tongue cancer SCC-4 cells	Reduced urokinase-plasminogen activator (u-PA)	Antimetastatic, Anticancer	[34, 72]

Vascular endothelial growth factor A	*VEGFA*	HUVECs	Prevention of VEGF expression	Tumour angiogenesis	[102]

Wee1-like protein kinase	*Wee1*	Gastric carcinoma SNU-5 cells	Increased expression of Wee1protein, G2/M phase arrest	Gastric cancer	[24]

**Table 2:** Berberine's target classification based on PANTHER.

Target names	Target gene symbol	PANTHER molecular function	Biological process	Pathway categories
Multidrug resistance protein 1 (Pgp, Pgp-170)	*ABCB1*	ATPase activity, coupled to transmembrane movement of substances, transmembrane transporter activity	Immune system process, extracellular transport, carbohydrate metabolic process, response to toxin	ATP-binding cassette (ABC) transporter

ATP-binding cassette sub-family G member 2	*ABCG2*	ATPase activity, coupled to transmembrane movement of substances, transmembrane transporter activity, anion channel activity	Immune system process, anion transport, lipid transport, oxygen and reactive oxygen species, metabolic process, lipid metabolic process, response to stress	N/A

Acetyl-coenzyme A carboxylase-*α*	*ACACA*	Other ligase	Gluconeogenesis, monosaccharide metabolism, fatty acid biosynthesis, coenzyme metabolism	N/A

*α*-Fetoprotein	*AFP*	Other transfer/carrier protein	Transport, mesoderm development, oncogenesis	N/A

Transcription factor AP-1	*AP-1*	DNA binding, transcription factor activity	Cell cycle, intracellular signaling cascade, nucleobase, nucleoside, nucleotide, and nucleic acid, metabolic process, cell cycle, signal transduction	Toll receptor signaling pathway, inflammation mediated by chemokine and cytokine signaling pathway, apoptosis signaling pathway, oxidative stress response, angiogenesis, TGF-beta signaling pathway, T-cell activation, B-cell activation, Ras Pathway, FAS signaling pathway, PDGF signaling pathway

Amyloid-*β* (A4) precursor protein (peptidase nexin-II, Alzheimer disease)	*APP*	Other signaling molecules	Other signal transduction, cell communication, other intracellular protein traffic	Alzheimer disease-amyloid secretase pathway, Alzheimer disease-presenilin pathway, blood coagulation, Alzheimer disease-presenilin pathway, Alzheimer disease-amyloid secretase pathway

Cyclic AMP-dependent transcription factor ATF-3	*ATF3*	DNA binding, transcription factor activity	Transcription factor activity, immune system process, neurological system process, induction of apoptosis, nucleobase, nucleoside, nucleotide, and nucleic acid metabolic process	Apoptosis signaling pathway

Bcl2 antagonist of cell death	*BAD*	N/A	N/A	PDGF signaling pathway, apoptosis signaling pathway, angiogenesis, PI3 kinase pathway, VEGF signaling pathway, interleukin signaling pathway

BCL2-associated X protein	*BAX*	Other signaling molecule	Induction of apoptosis, gametogenesis, hematopoiesis, cell cycle control, cell proliferation and differentiation, tumor suppressor	p53 pathway, apoptosis signaling pathway, Huntington disease

B-cell CLL/lymphoma 2	*BCL2*	Other signaling molecule	Inhibition of apoptosis, oncogenesis	Oxidative stress response, apoptosis signaling pathway

Apoptosis regulator Bcl-X	*BCL2L1*	Receptor binding	Gamete generation, induction of apoptosis, negative regulation of apoptosis, cell cycle, mesoderm development, hemopoiesis	Apoptosis signaling pathway

B-cell lymphoma 3-encoded protein	*Bcl-3*	N/A	Nucleobase, nucleoside, nucleotide, and nucleic acid metabolic process	Inflammation mediated by chemokine and cytokine signaling pathway

BH3-interacting domain death agonist p11	*BID*	N/A	N/A	Apoptosis signaling pathway, FAS signaling pathway

Baculoviral IAP repeat-containing protein 2 (anti-apoptosis factor c-IPA-1)	*BIRC2*	N/A	N/A	Apoptosis signaling pathway

Baculoviral IAP repeat-containing protein 3	*BIRC3*	N/A	N/A	Apoptosis signaling pathway

Baculoviral IAP repeat-containing protein 5 (Survivin)	*BIRC5*	N/A	N/A	Angiogenesis

Caspase 3, apoptosis-related cysteine peptidase	*CASP3*	Cysteine protease	Proteolysis, apoptosis	Huntington disease, FAS signaling pathway, apoptosis signaling pathway

Caspase 8, apoptosis-related cysteine peptidase	*CASP8*	Cysteine protease	Proteolysis, apoptosis	Apoptosis signaling pathway, FAS signaling pathway, Huntington disease

Caspase 9, apoptosis-related cysteine peptidase	*CASP9*	Cysteine protease	Proteolysis, apoptosis	Angiogenesis, apoptosis signaling pathway, FAS signaling pathway, VEGF signaling pathway, PI3 kinase pathway

Chemokine (C-C motif) ligand 2 (monocyte chemotactic protein-1)	*CCL2*	Nonreceptor serine/threonine, protein kinase	Protein phosphorylation, cell cycle control, mitosis	N/A

G2/mitotic-specific cyclin-B1	*CCNB1*	Protein binding, kinase activator activity, kinase regulator activity	Mitosis	Cell cycle, p53 pathway

G1/S-specific cyclin-D1	*CCND1*	Protein binding, kinase activator activity, kinase regulator activity	Spermatogenesis, mitosis	PI3 kinase pathway, cell cycle, Wnt signaling pathway

G1/S-specific cyclin-D2	*CCND2*	Protein binding, kinase activator activity, kinase regulator activity	Spermatogenesis, mitosis	PI3 kinase pathway, cell cycle

Cyclin E1	*CCNE1*	Kinase activator	Cell cycle control, mitosis, cell proliferation and differentiation	p53 pathway, cell cycle, Parkinson disease, p53 pathway feedback loops 2

G1/S-specific cyclin-E1	*CCNE1*	Protein binding, kinase activator activity, kinase regulator activity	Mitosis	p53 pathway, cell cycle, Parkinson disease, p53 pathway feedback loops 2

Interleukin-2 receptor alpha chain	*IL2RA/CD25*	Cytokine receptor activity	Immune system process, cell surface receptor-linked signal transduction, intracellular signaling cascade, cell-cell signalling, signal transduction, cell-cell signalling, cellular defense response	Interleukin signaling pathway

Platelet glycoprotein 4	*CD36/FAT*	Receptor activity	Macrophage activation, lipid transport, apoptosis, signal transduction, cell adhesion, lipid metabolic process, signal transduction, cell adhesion, cellular component, morphogenesis	N/A

Early activation antigen CD69	*CD69*	Receptor activity, receptor binding	B-cell-mediated immunity, natural killer cell activation, cellular defense response	Membrane-bound signaling molecule

M-phase inducer phosphatase 1	*CDC25A*	Hydrolase activity, acting on ester bonds, phosphatase activity	Phosphatase activity cell cycle, phosphate metabolic process, protein metabolic process, cell cycle	p53 pathway

Cdc42 effector protein 1	*CDC42EP1*	N/A	N/A	N/A

Cyclin dependant kinase 1/cell division control protein 2 homolog	*CDK1/CDC2*	Kinase activity	Immune system process, mitosis, intracellular signaling cascade, protein metabolic process, cell motion, mitosis, signal transduction, response to stress	p53 pathway

Cyclin-dependent kinase 2	*CDK2*	Nonreceptor serine/threonine protein kinase	Protein phosphorylation, cell cycle control, mitosis	p53 pathway, p53 pathway feedback loops 2

Cyclin-dependent kinase 4	*CDK4*	Nonreceptor serine/threonine protein kinase	Protein phosphorylation, cell cycle control, mitosis	N/A

Cell division protein kinase 6	*CDK6*	Kinase activity	Immune system process, mitosis, intracellular signaling cascade, protein metabolic process, cell motion, mitosis, signal transduction, response to stress	N/A

Cyclin-dependent kinase inhibitor 1 (p21)	*CDKN1A*	Protein binding, kinase inhibitor activity, kinase regulator activity	Cell cycle	Interleukin signaling pathway, p53 pathway feedback loops 2, p53 pathway

Cyclin-dependent kinase inhibitor 1B (P27/KIP1)	*CDKN1B*	Protein binding, kinase inhibitor activity, kinase regulator activity	Cell cycle	Interleukin signaling pathway

CASP8-and FADD-like apoptosis regulator subunit p12	*CFLAR/cFLIP*	Peptidase activity, protein binding, peptidase inhibitor activity	Apoptosis, protein metabolic process	Apoptosis signaling pathway, FAS signaling pathway

Inhibitor of NF-*κ*B kinase subunit alpha (I*κ*B kinase)	*CHUK(IKK)*	Kinase activity	Immune response, intracellular signaling cascade, protein metabolic process, signal transduction, response to stimulus	Interleukin signaling pathway, apoptosis signaling pathway, T-cell activation, toll receptor signaling pathway, B-cell activation

SDF-1-*α* (3–67) (SDF-1)	*CXCL12*	N/A	N/A	Axon guidance-mediated by Slit/Robo

Cytochrome c-1	*CYC1*	Reductase	Oxidative phosphorylation	FAS signaling pathway, ATP synthesis, Huntington disease

Cytochrome P450, family 2, subfamily C, polypeptide 9	*CYP2C9*	Oxygenase	Fatty acid metabolism, steroid metabolism, electron transport	N/A

Cytochrome P450, family 2, subfamily D, polypeptide 6	*CYP2D6*	Oxygenase	Other lipid, fatty acid and steroid metabolism, steroid metabolism, electron transport	Vitamin D metabolism and pathway

Cytochrome P450, family 3, subfamily A, polypeptide 4	*CYP3A4*	Oxygenase	Steroid hormone metabolism, electron transport	N/A

Dipeptidyl-peptidase 4 (CD26, adenosine deaminase complexing protein 2)	*DPP4*	Serine protease	Proteolysis, cell surface receptor mediated signal transduction, T-cell-mediated immunity	N/A

Epidermal growth factor receptor	*EGFR*	Kinase activity, transmembrane receptor protein tyrosine kinase activity, transmembrane receptor protein kinase activity, receptor binding	Female gamete generation, immune system process, negative regulation of apoptosis, cell cycle, cell surface receptor-linked signal transduction, intracellular signaling cascade, cell-cell signalling, cell-cell adhesion, protein metabolic process, cell motion, cell cyclesignal transduction, cell-cell signalling, dorsal/ventral axis specification, ectoderm development, mesoderm development, embryonic development, nervous system development	EGF receptor signaling pathway, cadherin signaling pathway

Receptor tyrosine-protein kinase erbB-2	*ERBB2/HER2*	Kinase activity, transmembrane receptor protein tyrosine kinase activity, transmembrane receptor protein kinase activity, receptor binding	Female gamete generation, immune system process, negative regulation of apoptosis, cell cycle, cell surface receptor linked signal transduction, intracellular signaling cascade, cell-cell signalling, cell-cell adhesion, protein metabolic process, cell motion, cell cyclesignal transduction, cell-cell signalling, dorsal/ventral axis specification, ectoderm development, mesoderm development, embryonic development, nervous system development	EGF receptor signaling pathway, cadherin signaling pathway

Mitogen-activated protein kinase 3	*ERK1/MAPK3*	Kinase activity	Immune system process, mitosis, cell surface receptor linked signal transduction, intracellular signaling cascade, carbohydrate metabolic process, protein metabolic process, cell motion, signal transduction, segment specification, ectoderm development, mesoderm development, embryonic development, nervous system development, response to stress	Apoptosis signaling pathway, Alzheimer disease-amyloid secretase pathway, B-cell activation, Ras pathway, interleukin signaling pathway, angiogenesis, T-cell activation, toll receptor signaling pathway, insulin/IGF pathway-mitogen activated protein kinase kinase/MAP kinase cascade, FGF signaling pathway, Parkinson disease, PDGF signaling pathway, inflammation mediated by chemokine and cytokine signaling pathway, VEGF signaling pathway, interferon-gamma signaling pathway, endothelin signaling pathway, angiogenesis, TGF-beta signaling pathway, integrin signalling pathway, EGF receptor signaling pathway

Mitogen-activated protein kinase 4	*ERK2/MAPK4*	Kinase activity	Immune system process, mitosis, cell surface receptor linked signal transduction, intracellular signaling cascade, carbohydrate metabolic process, protein metabolic process, cell motion, mitosis, signal transduction, segment specification, ectoderm development, mesoderm development, embryonic development, nervous system development, response to stress	Alzheimer disease-amyloid secretase pathway, interleukin signaling pathway, angiogenesis, VEGF signaling pathway, integrin signalling pathway

Ezrin	*EZR*	Structural constituent of cytoskeleton	Cellular component, morphogenesis	N/A

C/EBP homologous protein (CHOP) or growth arrest- and DNA damage-inducible gene 153 (GADD153) or DNA damage-inducible transcript 3	*GADD153/DDIT3*	N/A	N/A	Oxidative stress response

Glucagon-like peptide (GCG/GLP-1/GLP-2)	*GCG*	Receptor binding	Signal transduction, cell-cell signalling, carbohydrate metabolic process, lipid metabolic process, signal transduction, cell-cell signalling, cellular glucose homeostasis	Peptide hormone

Growth/differentiation factor 15 (NAG-1)	*GDF15*	Receptor binding	Female gamete generation, cell surface receptor linked signal transduction, signal transduction, ectoderm development, mesoderm development, skeletal system development, heart development, muscle organ development	TGF-beta signaling pathway

Hypoxia-inducible factor 1*α*	*HIF1A*	DNA binding, transcription factor activity	Nucleobase, nucleoside, nucleotide, and nucleic acid metabolic process, ectoderm development, nervous system development	Hypoxia response via HIF activation, VEGF signaling pathway, angiogenesis

Interferon-*β*	*IFNB1*	Receptor binding	Response to interferon-gamma, induction of apoptosis, negative regulation of apoptosis, cell surface receptor linked signal transduction, intracellular signaling cascade, cell-cell signalling, signal transduction, cell-cell signalling, cellular defense response	Toll receptor signaling pathway

Interleukin-1*β*	*IL1B*	Receptor binding	Immune response, macrophage activation, cell surface receptor linked signal transduction, cell-cell signalling, signal transduction, cell-cell signalling, response to stimulus	Inflammation mediated by chemokine and cytokine signaling pathway

Interleukin-6	*IL6*	Receptor binding	Immune system process, negative regulation of apoptosis, cell surface receptor linked signal transduction, intracellular signaling cascade, cell-cell signalling signal transduction, cell-cell signaling	Inflammation mediated by chemokine and cytokine signaling pathway, interleukin signaling pathway

Interleukin 8	*IL8*	Chemokine	Cytokine- and chemokine-mediated signaling pathways, calcium-mediated signalling, NF-kappaB cascade, ligand-mediated signalling, T-cell-mediated immunity, macrophage-mediated immunity, granulocyte-mediated immunity, angiogenesis, cell proliferation and differentiation, cell motility	Inflammation mediated by chemokine and cytokine signaling pathway, interleukin signaling pathway

Potassium voltage-gated channel subfamily H member 2	*KCNH2/HERG1*	Receptor activity, cation transmembrane transporter activity, voltage-gated potassium channel activity, cation channel activity, cyclic nucleotide-gated ion channel activity	Cation transport, signal transduction	Ligand-gated ion channel

Low-density lipoprotein receptor (familial hypercholesterolemia)	*LDLR*	Other receptor	Oogenesis	Alzheimer disease-presenilin pathway

Mitogen-activated protein kinase 8 (JNK)	*MAPK8*	Kinase activity	Immune system process, mitosis, cell surface receptor linked signal transduction, intracellular signaling cascade, carbohydrate metabolic process, protein metabolic process, cell motion, mitosis, signal transduction, segment specification, ectoderm development, mesoderm development, embryonic development, nervous system development, response to stress	Alzheimer disease-amyloid secretase pathway, Ras pathway, EGF receptor signaling pathway, Parkinson disease, angiogenesis, FGF signaling pathway, FAS signaling pathway, toll receptor signaling pathway, TGF-beta signaling pathway, PDGF signaling pathway, Huntington disease, integrin signalling pathway, T-cell activation, B-cell activation, interferon-gamma signaling pathway, oxidative stress response, apoptosis signaling pathway, integrin signalling pathway

Induced myeloid leukemia cell differentiation protein Mcl-1	*MCL1*	Receptor binding	Gamete generation, induction of apoptosis, negative regulation of apoptosis, cell cycle, mesoderm development, hemopoiesis	Apoptosis signaling pathway

Matrix metallopeptidase 1 (27 kDa interstitial collagenase)	*MMP1*	Peptidase activity	Protein metabolic process	Plasminogen activating cascade, Alzheimer disease-presenilin pathway, plasminogen activating cascade

Matrix metalloproteinase-16	*MMP16*	Peptidase activity	Protein metabolic process	Alzheimer disease-presenilin pathway

72 kDa type IV collagenase	*MMP2*	Metalloprotease, other extracellular matrix	Proteolysis	Alzheimer disease-presenilin pathway

Matrix metallopeptidase 9 (gelatinase B, 92 kDa gelatinase, 92 kDa type IV collagenase)	*MMP9*	Metalloprotease, other extracellular matrix	Proteolysis	Alzheimer disease-presenilin pathway, plasminogen activating cascade

Myc proto-oncogene protein	*MYC*	DNA binding, transcription factor activity	Induction of apoptosis, cell cycle, nucleobase, nucleoside, nucleotide, and nucleic acid, metabolic process, cell cycle	Oxidative stress response, p53 pathway feedback loops 2, Wnt signaling pathway, interleukin signaling pathway, PDGF signaling pathway

Arylamine *N*-acetyltransferase 1	*NAT*	Acyltransferase activity	Metabolic process	Acetyltransferase

Nuclear factor NF-*κ*B p50 subunit (NF-*κ*B)	*NFKB1*	DNA binding, transcription factor activity	B-cell-mediated immunity, negative regulation of apoptosis, intracellular signaling cascade, nucleobase, nucleoside, nucleotide, and nucleic acid metabolic process, signal transduction, cellular defense response	T-cell activation, B-cell activation, toll receptor signaling pathway, inflammation mediated by chemokine and cytokine signaling pathway, apoptosis signaling pathway

NF-*κ*B inhibitor-*α*	*NFKBIA*	Protein binding	Immune system process, intracellular protein transport apoptosis, intracellular signaling cascade, nucleobase, nucleoside, nucleotide, and nucleic acid metabolic process, signal transduction, response to stress	Apoptosis signaling pathway, toll receptor signaling pathway, inflammation mediated by chemokine and cytokine signaling pathway, T-cell activation, B-cell activation

Nucleophosmin (nucleolar phosphoprotein B23) and telomerase	*NPM1*	N/A	Nucleobase, nucleoside, nucleotide, and nucleic acid metabolic process	N/A

Nuclear receptor subfamily 3, group C, member 1 (glucocorticoid receptor)	*NR3C1*	Nuclear hormone receptor, transcription factor, nucleic acid binding	N/A	N/A

Poly(ADP-ribose) polymerase family, member 1	*PARP*	Glycosyltransferase	DNA repair, protein ADP-ribosylation, stress response	FAS signaling pathway

Proprotein convertase subtilisin/kexin type 9	*PCSK9*	Serine protease	Proteolysis	N/A

Urokinase-plasminogen activator	*PLAU*	Peptidase activity	Immune system process, signal transduction, protein metabolic process, cell motion, signal transduction, blood coagulation	Blood coagulation, plasminogen activating cascade

Peroxisome proliferator-activated receptor-*γ*	*PPARG*	Nuclear hormone receptor, transcription factor, nucleic acid binding	Monosaccharide metabolism, regulation of lipid, fatty acid, and steroid metabolism, mRNA transcription regulation, ligand-mediated signalling, stress response, developmental processes, cell proliferation and differentiation	N/A

Prostaglandin G/H synthase 2	*PTGS2/COX2*	Oxidoreductase activity	Immune system process	Endothelin signaling pathway, toll receptor signaling pathway, inflammation mediated by chemokine and cytokine signaling pathway

Ras-related C3 botulinum toxin substrate 1	*RAC1*	GTPase activity, protein binding	Intracellular protein transport, endocytosis, cell surface receptor linked signal transduction, intracellular signaling cascade, signal transduction	Axon guidance mediated by Slit/Robo, integrin signalling pathway, inflammation mediated by chemokine and cytokine signaling pathway, Huntington disease, axon guidance mediated by Slit/Robo, FGF signaling pathway, T-cell activation, axon guidance mediated by netrin, EGF receptor signaling pathway, inflammation mediated by chemokine and cytokine signaling pathway, cytoskeletal regulation by Rho GTPase, aAxon guidance mediated by semaphorins, cytoskeletal regulation by Rho GTPase, B-cell activation, Ras pathway

Rho-associated protein kinase 1	*ROCK1/RHO*	Kinase activity	Mitosis, intracellular signaling cascade, cell adhesion, protein metabolic process, cell motion, mitosis, signal transduction, cell adhesion, embryonic development	Inflammation mediated by chemokine and cytokine signaling pathway, cytoskeletal regulation by Rho GTPase

Transforming protein RhoA	*RHOA*	GTPase activity, protein binding	Intracellular protein transport, endocytosis, cell surface receptor linked signal transduction, intracellular signaling cascade, signal transduction	Axon guidance mediated by Slit/Robo, angiogenesis, heterotrimeric G-protein signaling pathway-Gq alpha; and Go alpha mediated pathway, axon guidance mediated by semaphorins, inflammation mediated by chemokine and cytokine signaling pathway, integrin signalling pathway, Ras pathway, cytoskeletal regulation by Rho GTPase, PDGF signaling pathway

Proto-oncogene tyrosine-protein kinase ROS	*ROS1*	Kinase activity, transmembrane receptor protein tyrosine kinase activity, transmembrane receptor protein kinase activity, receptor binding	Female gamete generation, immune system process, visual perception, sensory perception, negative regulation of apoptosis, cell cycle, cell surface receptor linked signal transduction, intracellular signaling cascade, cell-cell signalling, cell-cell adhesion, protein metabolic process, cell motion, cell cycle, signal transduction, ectoderm development, mesoderm development, embryonic development, nervous system development	N/A

Runt-related transcription factor 2	*RUNX2*	DNA binding, transcription factor activity	Mesoderm development, skeletal system development, hemopoiesis	N/A

Sucrase-isomaltase (Alpha-glucosidase)	*SI*	Hydrolase activity, hydrolyzing O-glycosyl compounds	Carbohydrate metabolic process, protein metabolic process	N/A

Processed sterol regulatory element-binding protein 2	*SREBP2*	DNA binding, transcription factor activity	Nucleobase, nucleoside, nucleotide and nucleic acid metabolic process, lipid metabolic process	Basic helix-loop-helix transcription factor

Tumor necrosis factor/tumor necrosis factor-*α*	*TNFA*	Tumor necrosis factor family member	Cytokine- and chemokine-mediated signaling pathways, ligand-mediated signalling, immunity and defense, induction of apoptosis	Wnt signaling pathway, apoptosis signaling pathway

Topoisomerase (DNA) I	*Top1*	DNA topoisomerase	DNA replication, general mRNA transcription activities	DNA replication

Cellular tumor antigen p53	*TP53*	DNA binding, transcription factor activity	Induction of apoptosis, cell cycle, nucleobase, nucleoside, nucleotide, and nucleic acid metabolic process, cell cycle	Apoptosis signaling pathway, Huntington disease, P53 pathway feedback loops 1, p53 pathway, p53 pathway by glucose deprivation, p53 pathway feedback loops 2, Wnt signaling pathway

Vascular endothelial growth factor A	*VEGFA*	Receptor binding	Immune system process, cell cycle, cell surface receptor linked signal transduction, intracellular signaling cascade, cell-cell signalling, cell cyclesignal transduction, mesoderm development, angiogenesis, response to stress	Angiogenesis, VEGF signaling pathway

Wee1-like protein kinase	*Wee1*	Kinase activity	Mitosis, protein metabolic process	Protein kinase

**Table 3:** Distribution of berberine's targets *in vitro* according to molecular functions.

PANTHER molecular function	Number of targets
Acyltransferase activity	1
Anion channel activity	1
ATPase activity, coupled to transmembrane movement of substances	2
Cation channel activity	1
Cation transmembrane transporter activity	1
Chemokine	1
Cyclic nucleotide-gated ion channel activity	1
Cysteine protease	3
Cytokine receptor activity	1
DNA binding	9
DNA topoisomerase	1
Glycosyltransferase	1
GTPase activity	2
Hydrolase activity, acting on ester bonds	1
Hydrolase activity, hydrolyzing O-glycosyl compounds	1
Kinase activator	1
Kinase activator activity	4
Kinase activity	11
Kinase inhibitor activity	2
Kinase regulator activity	6
Metalloprotease	2
Not classified	10
Non-receptor serine/threonine protein kinase	3
Nuclear hormone receptor	2
Nucleic acid binding	2
Other extracellular matrix	2
Other ligase	1
Other receptor	1
Other signaling molecule	3
Other transfer/carrier protein	1
Oxidoreductase activity	1
Oxygenase	3
Peptidase activity	4
Peptidase inhibitor activity	1
Phosphatase activity	1
Protein binding	10
Receptor activity	3
Receptor binding	12
Reductase	1
Serine protease	2
Structural constituent of cytoskeleton	1
Transmembrane transporter activity	2
Transcription factor	2
Transcription factor activity	9
Transmembrane receptor protein kinase activity	3
Transmembrane receptor protein tyrosine kinase activity	3
Tumor necrosis factor family member	1
Voltage-gated potassium channel activity	1

**Table 4:** Distribution of berberine's targets *in vitro* according to biological functions.

PANTHER biological functions	Number of targets
Angiogenesis	2
Anion transport	1
Apoptosis	6
B-cell-mediated immunity	2
Blood coagulation	1
Calcium-mediated signaling	1
Carbohydrate metabolic process	7
Cation transport	1
Cell adhesion	3
Cell communication	1
Cell cycle	11
Cell cycle control	5
Cell cycle intracellular signaling cascade	1
Cell cycle signal transduction	1
Cell motility	1
Cell motion	10
Cell proliferation and differentiation	3
Cell proliferation and differentiation	1
Cell surface receptor linked signal transduction	14
Cell surface receptor-mediated signal transduction	1
Cell-cell adhesion	3
Cell-cell signaling	9
Cellular component morphogenesis	2
Cellular defense response	4
Cellular glucose homeostasis	1
Coenzyme metabolism	1
Cytokine- and chemokine-mediated signaling pathways	2
Developmental processes	1
DNA repair	1
DNA replication	2
Dorsal/ventral axis specification	1
Ectoderm development	1
Ectoderm development	8
Electron transport	3
Embryonic development	7
Endocytosis	2
Extracellular transport	2
Fatty acid biosynthesis	1
Fatty acid metabolism	1
Female gamete generation	4
Gamete generation	2
Gametogenesis	1
General mRNA transcription activities	1
Gluconeogenesis	1
Granulocyte-mediated immunity	1
Heart development	1
Hematopoiesis	1
Hemopoiesis	3
Immune response	2
Immune system process	16
Immune system processMitosis	1
Immunity and defense	1
Induction of apoptosis	9
Intracellular protein transport	3
Intracellular signaling cascade	18
Ligand-mediated signaling	3
Lipid metabolic process	4
Lipid transport	2
Macrophage activation	2
Macrophage-mediated immunity	1
Mesoderm development	12
Metabolic process	1
Mitosis	4
Monosaccharide metabolism	2
mRNA transcription regulation	1
Muscle organ development	1
Not classified	9
Natural killer cell activation	1
Negative regulation of apoptosis	8
Nervous system development	7
Neurological system process	1
NF-*κ*B cascade	1
Nucleobase, nucleoside, nucleotide, and nucleic acid metabolic process	10
Oncogenesis	3
Other intracellular protein traffic	1
Other lipid, fatty acid and steroid metabolism	1
Other signal transduction	1
Oxidative phosphorylation	1
Oxygen and reactive oxygen species metabolic process	1
Phosphatase activity cell cycle	1
Phosphate metabolic process	1
Protein ADP-ribosylation	1
Protein metabolic process	17
Protein phosphorylation	3
Proteolysis	7
Regulation of lipid, fatty acid and steroid metabolism	1
Response to interferon-*γ*	1
Response to stimulus	2
Response to stress	8
Response to toxin	2
Segment specification	3
Sensory perception	1
Signal transduction	25
Skeletal system development	2
Spermatogenesis	2
Steroid hormone metabolism	1
Steroid metabolism	2
Stress response	2
T-cell-mediated immunity	2
Transcription factor activity immune system process	1
Transport	1
Tumor suppressor	1
Visual perception	1

**Table 5:** Distribution of berberine's targets *in vitro* according to pathway categories.

PANTHER pathway categories	Number of targets
Acetyltransferase	1
Alzheimer disease-amyloid secretase pathway	11
Alzheimer disease-presenilin pathway	14
Angiogenesis	11
Apoptosis signaling pathway	21
ATP synthesis	1
ATP-binding cassette (ABC) transporter	2
Axon guidance mediated by netrin	1
Axon guidance mediated by semaphorins	1
Axon guidance mediated by Slit/Robo	4
B-cell activation	7
Basic helix-loop-helix transcription factor	1
Blood coagulation	3
Cadherin signaling pathway	2
Cell cycle	4
Cytoskeletal regulation by Rho GTPase	3
DNA replication	2
EGF receptor signaling pathway	4
Endothelin signaling pathway	2
FAS signaling pathway	13
FGF signaling pathway	4
Heterotrimeric G-protein signaling pathway—Gq alpha- and Go alpha-mediated pathway	1
Huntington disease	9
Hypoxia response via HIF activation	1
Inflammation mediated by chemokine and cytokine signaling pathways	13
Insulin/IGF pathway-mitogen activated protein kinase kinase/MAP kinase cascade	1
Integrin signalling pathway	6
Interferon-gamma signaling pathway	2
Interleukin signaling pathway	10
Ligand-gated ion channel	1
Membrane-bound signaling molecule	1
Pathway unclassified	19
Oxidative stress response	5
p53 pathway	12
p53 pathway by glucose deprivation	1
p53 pathway feedback loops	1
P53 pathway feedback loops 1	1
p53 pathway feedback loops 2	4
Parkinson disease	3
PDGF signaling pathway	6
Peptide hormone	1
PI3 kinase pathway	4
Plasminogen activating cascade	8
Protein kinase	1
Ras Pathway	5
T-cell activation	7
TGF-*β* signaling pathway	4
Toll receptor signaling pathway	9
VEGF signaling pathway	7
Vitamin D metabolism and pathway	1
Wnt signaling pathway	4

**Table 6:** Comparison of clinical studies of berberine in diabetes patients.

Study type	Study subjects	Berberine dosage	Control treatment	Major findings	Side effects	Reference
Randomised, double-blind, placebo-controlled, multiple-center	Type 2 diabetes and dyslipidemia (*n* = 116)	0.5 g, b.i.d for 3 months	Placebo	Significantly reduced fasting and postload plasma glucose, HbA_1c_ Significantly reduced triglyceride, total cholesterol, and LDL-cholesterol	Mild to moderate constipation in 5 patients	[61]

Randomised, blinded, placebo-controlled	Type 2 diabetes (*n* = 36)	0.5 g, t.i.d for 3 months	Metformin (0.5 g t.i.d)	Significantly reduced FBG, PBG, and HbA_1c_ Significantly reduced plasma triglycerides	Transient gastrointestinal adverse effects. No liver or kidney damage	[14]
	Type 2 diabetes poorly controlled (*n* = 48)	0.5 g, t.i.d for 3 months	Existing anti-diabetic treatment	Lowered FBG and PBG Significantly decreased HbA_1c_ Significantly reduced fasting plasma insulin and HOMA-IR		

Randomised	Type 2 diabetes (*n* = 97)	1 g/day for 2 months	Metformin (1.5 g/day); rosiglitazone (4 mg/day)	Significantly reduced FBG, HbA_1c_, and triglycerides Serum insulin level was declined significantly (*P* < 0.01), increased insulin sensitivity in peripheral tissues Significantly elevated surface expression of InsR by 3.6-fold	No adverse events	[62]
	Type 2 diabetes with chronic hepatitis C virus infection (*n* = 35)	1 g/day for 2 months	N/A	Significantly reduced FBG and triglyceride levels Reduced the elevated ALT and aspartate aminotransferase levels		

b.i.d: twice daily; t.i.d: three times daily; FBG: fasting blood glucose; HOMA-IR: homeostasis model of assessment—insulin resistance; PBG: postprandial blood glucose.

## Abbreviations

Bax: BCL2-associated X protein
FBG: Fasting blood glucose
GLP-1: Glucagon like peptide 1
HbA_1c_: Hemoglobin A_1c_
InsR: Insulin receptor
PBG: Postprandial blood glucose.
